# Cisatracurium Retards Cell Migration and Invasion Upon Upregulation of p53 and Inhibits the Aggressiveness of Colorectal Cancer

**DOI:** 10.3389/fphys.2018.00941

**Published:** 2018-07-31

**Authors:** Iddrisu B. Yabasin, Jaceline G. P. Sanches, Mohammed M. Ibrahim, Jin Huidan, Walana Williams, Zhi-Li Lu, Qingping Wen

**Affiliations:** ^1^Department of Anesthesiology, First Affiliated Hospital of Dalian Medical University, Dalian, China; ^2^Department of Pathology and Forensics, Dalian Medical University, Dalian, China; ^3^Department of Microbiology and Immunology, Dalian Medical University, Dalian, China; ^4^Department of Ophthalmology, First Affiliated Hospital of Dalian Medical University, Dalian, China

**Keywords:** cisatracurium, migration, invasion, colorectal cancer, p53

## Abstract

Colorectal cancer (CRC) is reported to be the third and fourth, most diagnosed and cause of cancer associated deaths respectively. In 2012 for instance, about 1.4 million new cases were reported, and approximately 700,000 deaths recorded. Survival from CRC is dependent on the stage at which it is diagnosed coupled with appropriate surgical and medical intervention. Cisatracurium is widely used for skeletal muscle relaxation during abdominal surgeries, including bowel and colon surgeries. Recent studies reported that cisatracurium inhibits progression of human cancer cells, however, the mechanisms leading to the inhibition are yet to be completely understood. To elucidate mechanisms resulting particularly in tumor cell growth and metastasis, we developed *ex vivo* and in *in vivo* xenograft models of CRC. Cisatracurium caused upregulation of p53 and its down-stream genes and proteins known to regulate proliferation and metastasis *in vitro* and *in vivo*. Genomic analyses of CRC following cisatracurium treatment revealed moderate to high DNA damage, while functional analyses demonstrated significant tumor cells growth regression, as well as repression of migration and invasion. Importantly, cisatracurium increased E-Cadherin and CALD-1 but decreased SNAI-1 and SLUG levels *in vitro* and in *vivo*. Together, the findings demonstrate that elevation of p53 upon cisatracurium-induced genomic injury, represent a potential mechanism by which cisatracurium result in the suppression of CRC progression and metastasis.

## Introduction

Globally, CRC is rated third and fourth as commonly diagnosed and cause of cancer related deaths respectively. In 2012 alone, an estimated 1.4 million new cases were diagnosed, and about 700,000 deaths recorded (Ferlay et al., 2013). These projections are expected to rise by 60% by the year 2030, resulting in over 2.2 million cases, and 1.1 million deaths. The burden of CRC remains relatively huge in the developed world where more than two-thirds of global cases and an estimated 60% of CRC associated deaths occur (Ferlay et al., 2013). In the past few decades, both low and middle-income countries, have been experiencing surge in both incidence and mortality of CRC cases (Center et al., 2009).

It has been established that the outcome of CRC treatment depends on a number of factors, key among them are the stage of the cancer and the degree of metastasis (Gatta et al., 2000; Barton et al., 2006; Richards, 2009). Undoubtedly, continuous accelerated cancer cell proliferation and metastases are the reason for the poor outcome of most cancer cases, and the main cause of about 90% of cancer-related deaths (Weigelt et al., 2005).

Uncontrollable tumor growth is among the significant prognostic elements in the evolution of both primary and secondary tumors. The cell cycle is a well-organized intracellular program that maintains normal cell division and growth, however, deregulation of cell cycle machinery occurs frequently in significant number human malignancies, not only for abnormal cell growth but also for elevation of aggressive metastatic features (Massague, 2004; Besson et al., 2008).

The interplay among the distinct cyclin-dependent kinases (CDKs) and their corresponding cyclins is fundamentally the mechanism by which the cell cycle advancement is maintained. Cyclins, D1 and E1 are type of cyclins known to play key role in ensuring smooth transition of G1-S phase (Sherr, 1994). Cyclin Dependent Kinase 4 and 6 (CDK4/6) unite with Cyclin D1, while Cyclin Dependent Kinase 2 (CDK2) couples with cyclin E (Koff et al., 1992; Bates et al., 1994). The activity of the complexes resulting from coupling of CDKs and cyclins are strictly controlled by minute proteins termed cyclin dependent kinase inhibitors (CDKIs). p16, p18, and p19 are affiliates of INK4 clan. They preferentially interact with CDK4/6, in order to impede their association with members of cyclins D family (Serrano et al., 1993; Guan et al., 1994; Hirai et al., 1995). On the other hand, p21CIP1/WAF1 (p21) and p27KIP1 constitute the family of KIP (el-Deiry et al., 1993; Harper et al., 1993; Toyoshima and Hunter, 1994). They are known to possess high affinity for complexes arising from coupling of cyclin E and CDK, and cyclin A and CDK, but a number of studies demonstrated that they also couple with the complexes of cyclin D-CDK (Yu et al., 1998; Sherr and Roberts, 1999).

Previous reports revealed that the incidence of excessive p21 down-regulation and cyclin-D up-regulation are frequently observed in human cancers, particularly in melanomas, cervical and prostate carcinomas, and that these abnormal expressions appear to be associated with elevated tumor spread and poor treatment outcome (Abbas and Dutta, 2009; Musgrove et al., 2011).

Cancer metastasis have been reported to correlate inversely with apoptosis. The intrinsic apoptosis pathway encompasses several suicidal stimuli, one of which is genotoxic stress. These stimuli cause the outpour mitochondrial associated proteins, particularly Cytochrome C. Cytochrome C mediates apoptosome formation, resulting in the activation of the initiator, caspase-9. Other proteins which control the release of Cytochrome C include the B-cell lymphoma 2 (BCL-2) family, consisting of apoptotic repressors BCL2 and BCL-XL, and apoptotic promoters BAX and BH3 (Mehlen and Puisieux, 2006).

During genotoxic stress, the tumor suppressor p53 is activated with a corresponding transcription of BAX and the BAD (BCL2-antagonist of cell death). Alternatively, Caspase-2 is activated through p53- induced protein with a death domain (PIDD), culminating into the discharge of Cytochrome C. The induction of apoptosis is critical as it delays cancer proliferation, thereby retarding the progression of onco-genes conversion and generally restricting tumorigenesis (Yin et al., 1997; Lowe et al., 2004).

It has been established that inhibition of apoptosis is associated with metastasis. Experimentally, an estimated 0.01% of invasive clonal malignant cells introduced into the circulatory system could produce invasive foci (Liotta et al., 1978; Mehlen and Puisieux, 2006). This observation has been supported by other reports where tumorigenesis and accelerated tumor growth was found to be associated with either inhibition of BAX, TP53 as well as death-associated protein kinase (DAPK), or activation of BCL2 (Um et al., 2004). Similarly, breast cancer cells with elevated expression of BCL2 exhibit high level lung metastases in nude mice (Mehlen and Puisieux, 2006). Also, Lewis et al. (2005), have demonstrated that hepatocellular carcinoma with malfunctioned p53 significantly induces metastasis to the lung in their mouse model study. In conformity with previous reports, breast cancers metastasis is enhanced in transgenic mice containing HCCR-2, a human cervical cancer oncogene, that encrypts a negative regulator of p53 (Ko et al., 2004). Conversely, forcefully expressing DAPK, an enhancer of cell death which expression is usually less in invasive human carcinomas (Esteller, 2003), mitigates the metastatic aggressiveness of cancer cells (Inbal et al., 1997).

Following diagnoses of colon and rectal cancers, the treatment regimens usually involve resection of affected sites and associated lymph nodes and vessel, and administration of chemotherapy to patients prone to recurrence (Brenner et al., 2014). However, 30–50% of patients who experience surgical resection suffer recurrent of the disease (Schmoll et al., 2012). This suggests that, optimizing surgery and anesthesia strategy is critical, and could hold the key to reducing CRC recurrence following surgical resection. Excising colorectal tumors requires administering anesthetic agents for adequate muscle relaxation. Cisatracurium is a non-depolarizing muscle relaxant commonly used for surgeries requiring effective skeletal muscle paralysis. It has been demonstrated in previous studies that cisatracurium promotes apoptosis in human CRC cells through the p53 intrinsic apoptotic pathway (Yabasin et al., 2017). Backed by available evidence, it stands to reason that it may as well-repress the metastatic potential of CRC.

In this work, we aimed to investigate the role of cisatracurium in CRC metastasis *ex vivo* and *in vivo*. Herein we have demonstrated that overexpression of p53, accompanied with gain-and-loss of expression of downstream transcription factors such as E-Cadherin, CALD1, SNAI-1 and SLUG, following the exposure of CRC cells to cisatracurium results in the suppression of CRC cells/tumor growth as well as significant regression of migration and invasion.

## Materials and Methods

### Materials

Cisatracurium (Nimbex GlaxoSmith-Kline, England); HCT116 cell line from American Type Culture Collection (ATCC), United States; Cell Counting Kit-8 (CCK-8), Japan; FBS, PAA, Australia; Rowell Park Memorial Institute Medium-1640 (RPMI-1640), 50 units/mL of penicillin and streptomycin, Gibco, United States were obtained for cell culture and subsequent experiments.

### Cell Culture

The human CRC cell line HCT116, was routinely grown and maintained in RPMI with 10% FBS, 50 IU/ml and 50 μg/ml penicillin and streptomycin at 37°C in a humidified incubator with 5% CO_2_. At 80% confluence, cells were detached with trypsin. After centrifugation at 1500 rpm for 5 min, cells were collected and distributed in a medium containing 10% FBS and antibiotics. Subsequently, cells were quantified and used for further functional investigations. The maintenance medium of culturing cells was replaced at least twice a week.

### Cell Growth Assay

CCK-8 was used to measure proliferation response to cisatracurium treated and unentreated HCT116 cells in accordance with the manufacturer’s protocol. Concisely, 3 × 10^3^ cells were seeded into three identical wells of 96-well plate containing 0.1 ml of RPMI 1640 growth medium, 10% FBS and the antibiotics mentioned above. After cells had attached after 24 h in an incubator under standard conditions, they were exposed to cisatracurium at 10 or 20 μM for 24, 48, and 72 h. At the end of the above stated exposure periods, 0.01 ml of a calorimetric agent, CCK-8 was placed in each of the wells containing the control samples as well as those containing cisatracurium exposed samples. The samples were kept in an incubator for 60 min, after which the absorbance was measured with MultiskanGo Spectrophotometer (United States) at 450 nm optical density (OD). Suppression of cell proliferation was determined by the following mathematical relation: [(Mean absorbance of control – Mean absorbance of exposed)/Mean absorbance of control) × 100]. The experiment was conducted at three consecutive times.

### Clonogenic Assay

To determine the impact of cisatracurium on the clonogenicity of HCT116 cells, colony formation experiment was employed. 500 HCT116 cells were placed in three identical wells of 6-well plate with medium containing 10% FBS. The 6-well plates were placed in an incubator overnight to enable cells adhere to the base of the plate. Cells were treated with cisatracurium (10 or 20 μM) for 48 h, after which the media in control and treated samples were changed with media without cisatracurium. The samples were then placed in an incubator for 1 week for the formation of clones. Proliferation of cells was stopped by a mixture of 25% acetic acid in ethanol and colored with 1% crystal violet. Clones consisting 50% cells were counted. The assay was conducted at three consecutive times.

### Comet Assay

The most common cause of functional p53 over-transcription and over-translation is DNA damage. This phenomenon often occurs to allow for injured DNA to be fixed. Due to significant upregulation of p53 at both mRNA and protein levels in cisatracurium-treated cells, genotoxic assessment of cisatracurium-treated cells was conducted using comet assay to determine whether the significant rise in functional p53 could be attributed to DNA damage. In brief, untreated and cisatracurium-treated cells cultured for 48 h were redistributed in 1x PBS to a density of 1 × 10^6^ per 1000 μl. The untreated and cisatracurium-treated cells were combined with molten low melting agarose gel (LMA gel) at ratio of 1:10 (thus 50 μl of cells in 1× PBS at 1 × 10^6^/ml 500 μl of LMA agarose at 37°C). The assay was carried out as previously performed by Cordelli et al. (2007). LMA gel containing cells that are sandwiched by normal melting agarose (NMA) gel on slices were placed flat in the refrigerator at 4°C for 10 min. Slides were immersed in lysis buffer at 4°C overnight. Incubating slides in 4°C enhances sensitivity. After overnight incubation in lysis buffer, residual buffer on the slides were removed and submerged in newly constituted alkaline unwinding solution (pH > 13) for 20 min at 4°C in a refrigerator. Following 20 min incubation, slides were placed in electrophoresis tray containing alkaline solution and covered with slide tray overlay. The power supply was set to 25 V and voltage applied for 30 min. After 30 min, excess electrophoresis solution was gently drained, carefully immersed thrice in 0.4 mmol Tris-HCL pH 7.5 for 10 min each. Slides were dried at 37°C 10–15 min. Drying brings in a single plain to facilitate observation. Slides were then incubated in propidium iodide (PI) for 10 min in darkness. Slides were viewed with fluorescence microscope (Olympus IX73, Olympus Corporation, Tokyo, Japan) 515–560 nm wave length. Nuclear DNA and migrating DNA (comet tail) labeled with PI appeared red under fluorescence microscope. 50 cells were selected at random from the untreated and cisatracurium-treated groups slides and examined using computerized image analysis system (Delta Sistemi, Rome, Italy). Damaged DNA is classified into 5 scales according to the quantity of DNA in tail (tail intensity) as follows. Class 0: <5% no damage; class 1: 5–20% mild damage; class 2: 20–40% moderate damage; class 3: 40–95% high damage, and class 4: >95% severe damage. The assay was conducted at three consecutive times.

### Trans-Well Migration and Invasion

In order to investigate the impact of cisatracurium on CRC cells migration and invasion, trans-well experiments were conducted. A seeding density of 2 × 10^4^ cells were placed in the inserts (pore size 8 μm) of the 24 well plate (Corning, NY, United States) with 200 μl FBS free RPMI 1640. With regards to invasion, the base of the trans-well inserts was overlaid with extracellular matrix (ECM) gel (BD Biosciences, United States) to represent a membrane. The wells beneath the inserts contained chemoattractant, consisting of 700 μl RPMI 1640 with 20% FBS. The trans-well culture plates were kept in an incubation with standard conditions for a period of 16 h. After 10 min incubation in ethanol, cells were colored with 1% crystal violet for 20 min, and the inserts were gently cleaned and dried. The migration and invasion were assessed by observing the cells trapped to the back of the trans-well base using florescent microscope (Olympus IX73; Olympus Corporation, Tokyo, Japan). Five field views of migrated or invaded cells were randomly photographed. Migrated and invaded cells in each of the five field views were computed and averaged.

### Scratch Wound Healing Experiment

In our bid to further probe the effect of cisatracurium on CRC cells’ migratory potential, scratch wound healing experiment was employed. In summary, 2 × 10^5^ HCT116 and cisatracurium-treated HCT116 cells were maintained in 6-well plate. At 80% confluence of growth, cells were carefully wounded with a 200-μl pipette tip. The wells were cleansed two times with PBS to ensure that scratched debris or cells were washed off. Complete medium with or without cisatracurium was added and cultured at 37°C in 5% CO_2_ humidified incubator.

The rate of wound closure, which signifies cell migration, was monitored by imaging at time-points 0, 24 and 48 and 72 h using inverted fluorescent microscope with a digital camera incorporated. Duplicate wells for control and the treatment groups were examined. The experiment was repeated for three consecutive times.

### qRT-PCR Analysis

After 72 h of cells exposure to cisatracurium, total RNA was harvested with Trizol reagent (Invitrogen, United States) from the control and treated cells (10 or 20 μM), and cDNA constructed with oligonucleotide dT primer. Using qRT-PCR technique and SYBER green, transcription of the genes required were determined. With β-actin as internal control, fold change in mRNA relative to group control was computed using the formula 2^−ΔΔC_T_^. **Table 1** shows the sets of primers used for targeted genes in this study. Mx 3005P qRT-PCR instrument (Agilent Technologies, Germany) was used for the polymerase chain reaction. The machine was programmed to achieve 45 cycles of denaturation at 95°C for 0.5 min, annealing at 55°C for 0.5 min, and extension at 72°C for 0.5 min.

**Table 1:** Nucleotide sequence of primers used in qRT-PCR.

Gene	Primers	
	Forward	Reverse
CD1	TGTCCTACTACCGCCTCACA	CTTGGGGTCCATGTTCTGCT
p53	ACCTATGGAAACTACTTCCTGAAA	CTGGCATTCTGGGAGCTTCA
p21	GCGACTGTGATGCGCTAATG	GAAGGTAGAGCTTGGGCAGG
SNAI-1	ATTCTGTGGGCGTTGCTTTG	GTGACGCTGACGGACTTGTA
SLUG	GCGTTTTCCAGACCCTGGTT	CTTCATGCAAATCCAACAGCCA
E-Cad	GTCTCCTCTTGGCTCTGCC	TCGACCGGTGCAATCTTCAA
CALD1	GAGCATGCCTAGGGAATGACA	GAGGCGGTGGTATGCATTGT
β-Actin	ATGGAATCTTGCGGCATCCA	TTCTGCATCCTGTCGGCAAT

### Immunoblotting Analysis *ex Vivo* and *in Vivo*

Protein expression in control, cisatracurium-treated HCT116 cells as well as animal tissue samples was evaluated by immunoblotting technique using the required cell lysates and homogenized tissue samples as previously described (Yabasin et al., 2017). At about seventy percent growth, cells from control and those that had 72 h cisatracurium treatment were lysed with RIPA buffer, PMSF and 100x anti-protease cocktail and the supernatant used for the western blot evaluation. Using BCA kit and MultiskanGo Spectrophotometer (United States), the concentration of protein in both cell lysates and homogenized tumor samples were measured. Protein segregation was achieved by electrophoresis technique using a loading concentration of 20 μg. Following successful separation, proteins were transferred from SDS-PAGE to PVDF membrane-like paper (Invitrogen, United States). Isolation of individual proteins was achieved by primary and secondary antibodies; anti-CD1 (Boster, China), anti-p53 (Boster, China), anti-p21 (Boster, China), SNAI-1 (Proteintech, China), SLUG (Proteintech, China), E-Cadherin (Proteintech, China), CALD1 (Proteintech, China), and anti-β-actin (Boster, China) are the antibodies that were used in this work. The secondary antibodies were either rabbit anti-mouse (Boster, China) or mouse anti-rabbit (Boster, China) to commensurate the primary antibodies used. β-Actin was used as internal control, and Li-Cor Odyssey Infrared Imaging System (Version 3.0 software) was used to view and take images of protein bands.

### Immunofluorescence Staining

As previously explained (Yang et al., 2017), immunocytochemistry was carried out following exposure of HCT116 cells to cisatracurium. In summary, HCT116 cells were incubated in PBS containing 4% para-formaldehyde at 25°C for 20 min. For permeabilization, HCT116 cells were further maintained in PBS containing 0.5% Triton X-100 at 4°C for 10 min. After blocking using 3% BSA reagent, cells were exposed to primary antibodies for SLUG, SNAI1, E-Cadherin, and CALD1 (Proteintech). FITC conjugated secondary antibody (Invitrogen) was added and then stained with 1 μg/ml of DAPI for 60 s. Images of samples were visualized and captured using a fluorescence microscope (Olympus BX83, Japan).

### Mouse Xenograft Assay

Twelve 6–8-week-old NOD/SCID mice, weighing 18–25 g were purchased from the Dalian Medical University’s animal facility. Research protocols were reviewed and approval granted by Dalian Medical University’s Animal Research Ethics Committee. Mice were grouped into two by randomization. Each group had six mice. Mice in group 1 (control) were inoculated with untreated cells whiles that of group 2 were injected with cells that were treated with 20 μM cisatracurium for 72 h. The right hind leg of the mice was used as the site for subcutaneous inoculation. All animals received cells suspension of 100 μl.

The development and growth of tumor were closely monitored and measured at 7 days intervals, for four consecutive times. As previously described, the length and width of tumors were used to compute the volumes of tumors using the following mathematical relation: [(length (mm) × width (mm)2)]/2 (Workman et al., 2010). On the last day of the fourth week, mice were humanely sacrificed by euthanasia to allow for dissection. This was done in accordance with previously described protocol (Workman et al., 2010). Tumors were harvested, weighed, and recorded. Some of the tumor tissues were preserved for western blot analysis.

### Statistical Analysis

Data analysis was achieved with GraphPad Prism 6 statistical analysis software (United States). Where appropriate independent *t*-test or one-way analysis of variance was used to evaluate the differences in outcome between control and treatment groups in each experiment. Data are shown as Mean ± SEM. *p* < 0.05 is considered statistically significant.

## Results

### Cisatracurium Upregulates the Protein and mRNA Levels of p53 in CRC

**FIGURE 1:**
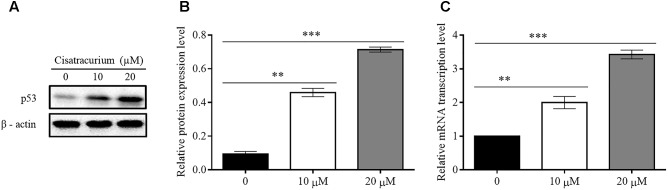
**(A–C)** Cisatracurium (10 or 20 μM) increases the expression of p53 in CRC cells. **(A)** Assessment of p53 protein expression level in HTC116 cells. **(B)** Bar chart of quantified p53 protein expression in HTC 116 cells. **(C)** Bar chart of quantified p53 mRNA levels in HTC116 cells. Data are presented as Mean ± SEM (*n* = 3). Statistical significant differences in protein and mRNA of colon cancer cells were observed [^∗∗^*p* < 0.01 and ^∗∗∗^*p* < 0.001 versus control (0)].

Based on a recent study in which cisatracurium was found to induce G1 arrest and substantially increased apoptosis in human CRC, it was suggested that cisatracurium could be causing DNA damage, resulting in increased p53 activation and altering the regulation of its downstream transcription factors that are key to tumor cell survival, growth, and metastasis. To investigate this observation, HCT116 cells were exposed to 10 and 20 μM cisatracurium and used for western blot and qRT-PCR analysis. The results indicate that cisatracurium increases mRNA and protein levels of p53 in HCT116 cells compared to control (**Figures 1A–C**). Cisatracurium increased the level of p53 protein expression by fivefold of the untreated cells for cells exposed to 10 μM and by sevenfold of the untreated cells for cells exposed to 20 μM (**Figure 1B**). On the other hand, mean fold change in mRNA level of p53 in the HCT116 cells exposed to cisatracurium 10 or 20 μM were approximately 2 and 3.5 folds of the untreated HCT116 cells respectively (**Figure 1C**). The magnitude of HCT116 cells sensitivity to cisatracurium and the cisatracurium-induced upregulation of p53 are concentration dependent.

### Cisatracurium Induces DNA Damage in CRC

**FIGURE 2:**
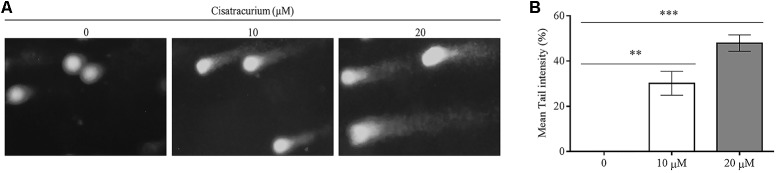
**(A,B)** Cisatracurium induces DNA damage in HTC cells. **(A)** Genotoxic assessment illustrates typical comet images from HTC116 cells treated with cisatracurium. It indicates different levels of damage: control (0), undamaged (no tail DNA); 10 and 20 μM shows increasing levels of damage. The number of cells scored in each measured concentration was 50. **(B)** Bar chart of mean tail comet in percentage [^∗∗^*p* < 0.01 and ^∗∗∗^*p* < 0.001 versus untreated (0)].

To determine the possible reason for the surge in p53 gene transcription and translation in HCT116 cells, a genotoxic assessment of HCT116 cells treated with cisatracurium was conducted using comet DNA assay. The result as illustrated in **Figure 2A**, indicates increasing comet tail with increasing cisatracurium concentration. Unlike the cisatracurium treated cells, the control (untreated) cells did not develop comet. DNA intensity was significantly higher in HCT116 cells exposed to cisatracurium than unexposed cells (**Figure 2B**). Moderate DNA damage (30%) was observed in cells that were treated with 10 μM cisatracurium, whiles higher DNA damage (48%) was observed in cells treated with 20 μM cisatracurium.

### Cisatracurium Retards CRC Cells Growth

**FIGURE 3:**
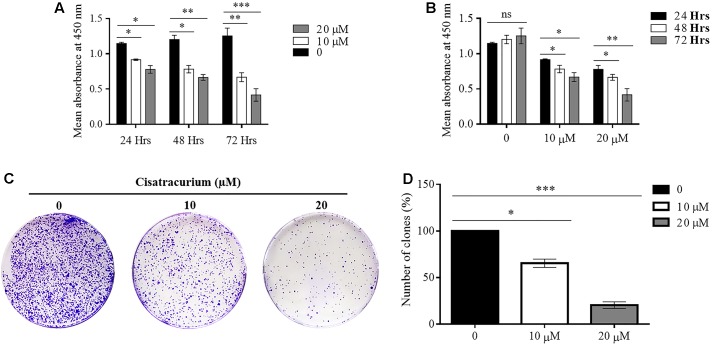
**(A–D)** Cisatracurium suppresses HCT116 growth and colony formation. Following exposure to cisatracurium (10 or 20 μM), cell growth was assessed using CCK-8 assay and analyzed by MultiscanGo photospectrometer. **(A)** Time dependent assessment of proliferation of untreated CRC cells (0) and cisatracurium-treated cells. **(B)** Cisatracurium concentration dependent assessment of CRC cells proliferation. Hrs represent in **(A,B)** represent hours. Data are presented as Mean ± SEM (*n* = 3). Statistical significant differences in colon cancer cells growth suppression [^∗^*p* < 0.05, ^∗∗^*p* < 0.01, and ^∗∗∗^*p* < 0.001 versus control (0) and 24 h respectively] were observed. **(C)** HCT116 colonies formed in control (0) and treatment groups (10 and 20 μM). **(D)** Graphical presentation of percentage of colonies formed in untreated and treatment groups. Data are expressed as Mean ± SEM (*n* = 3). ^∗^*p* < 0.05 and ^∗∗∗^*p* < 0.001 versus control.

In a typical *ex vivo*, cell growth experiment was done with CCK-8 kit. HCT116 cells treated with 10 and 20 μM cisatracurium had their growth rates decreased substantially, dependent on both concentration and duration of exposure relative to the control (**Figures 3A,B**). Following 24 h cisatracurium treatment, cells in the treatment groups (10 or 20 μM) had decreased by 16 and 27% respectively compared with control. The repression in growth rate in treatment groups 10 and 20 μM relative to control were 36 and 39% respectively at 48 h. At 72 h of treatment, the inhibition rate further increased accordingly by 45 and 67% compared to control. With regards to the rate of CRC cell growth based on time, similar patterns were observed. HCT116 cells in the control (0) had increased in growth rate at 48 and 72 h compared with 24 h. However, the increase in proliferation rate observed were comparable among the three time-points (*p* > 0.05). When cells were exposed to 10 and 20 μM cisatracurium, survival rate decreased significantly in the 48 and 72 h time points as compared to 24 h (^∗^*p* < 0.05 and ^∗∗^*p* < 0.01 versus 24 h) (**Figure 3B**).

To probe further, the effect of cisatracurium on the survival and proliferation of HCT116 cells was assessed using colony formation experiment. The results indicate significantly fewer clones in the treated cells compared with control (^∗^*p* < 0.05 and ^∗∗∗^*p* < 0.001) (**Figures 3C,D**). The cells incubated in 10 and 20 μM cisatracurium showed lesser percentage clones than that of the control (68 and 22%, respectively). Moreover, cells treated with 20 μM cisatracurium could not develop into dense clones compared with cells in 10 μM cisatracurium and control groups (**Figures 3C,D**). Also, many cells in the treatment groups had no physical contact with one another, instead they were loosely organized, particularly in the 20 μM cisatracurium treated cells (**Figure 3C**).

### Cisatracurium Impedes the Metastatic Ability of CRC

**FIGURE 4:**
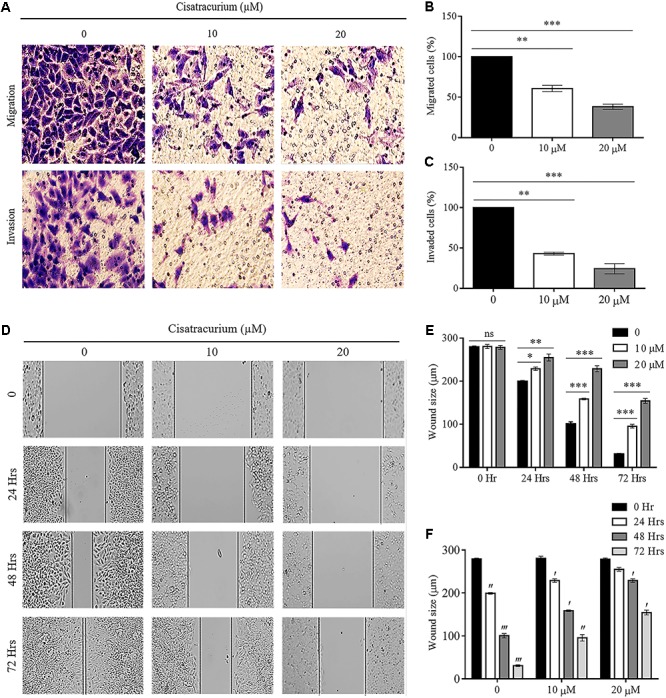
**(A–F)** Cisatracurium inhibits migration and invasion in CRC cells. **(A)** Representative pictures of migrated and invaded HTC116 cells after transwell migration and invasion assays. **(B)** Bar chart of percentage of cells migrated. **(C)** Bar chart of percentage of cells invaded. **(D)** Representative pictures of wound area following scratched wound healing assay. **(E)** Cluster bar chart of wound area in micrometers (μm) at various cisatracurium concentrations. **(F)** Cluster bar chart of wound area in μm at various time-points. Data are expressed as Mean ± SEM (*n* = 3). ^∗^*p* < 0.05, ^∗∗^*p* < 0.01, and ^∗∗∗^*p* < 0.001 versus untreated (0). ′*p* < 0.05, ^′′^*p* < 0.01, and ^′′′^*p* versus 0 Hr.

The results observed about the migration ability of the cells using transwell chamber without ECM lining gave a palpable trend based on the concentrations of cisatracurium. The differences in migration observed in the control and treated cells were significant. As indicated in **Figures 4A–C**, significant reductions in the number of HCT116 cells migrating through the transwell membrane with increasing concentration of cisatracurium was observed. This reduction was more pronounced in the 20 μM concentration where they were about threefold less than the migration observed in the control (*p* < 0.05). With regards to the invasion assay, it was observed that the number of HCT116 cells in the control which invaded the ECM membrane were far more than that of the cisatracurium-treated HCT116 cells that invaded the ECM. The significant reduction in the metastatic potential of HCT116 cells observed following the transwell migration and invasion assays further supports the inhibitory effect of cisatracurium.

Furthermore, scratch wound healing assay results add credence to the impact of cisatracurium on HCT116 cells’ migration and invasion observed in the trans-well assay (**Figures 4D–F**). The assay determines the magnitude of HCT116 cells motility based on the closure of wound area. Results of scratch wound healing assay in this study showed comparable wound area in control and treatments groups (10 and 20 μM) at time points 0 h (**Figures 4D,E**). However, there was significant inhibition of wound closure in the treatment groups (10 and 20 μM) compared with control or untreated HCT116 cells following 24, 48, and 72 h of incubation (**Figures 4D,E**) (*p* < 0.05). With the exception of time-point 0 and 24 h in cells treated with 20 μM, similar trends regarding wound size in time-point 0 compared with 24, 48, and 72 h in the control and cisatracurium-treated HCT116 cells were observed (*p* < 0.05) (**Figures 4D,F**).

**FIGURE 5:**
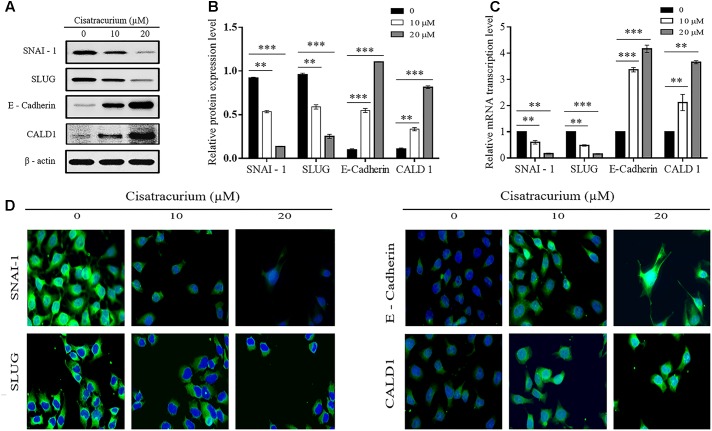
**(A–D)** Cisatracurium alters migration and invasion regulatory genes transcription and protein expression levels in HCT116 cells. **(A)** Representative densities of SNAI-1, SLUG, E-Cadherin, CALD1, and β-actin proteins following western blot experiment. **(B,C)** Cluster bar charts of SNAI-1, SLUG, E-Cadherin, and CALD1 proteins expression and mRNA transcription levels of untreated and cisatracurium-treated (10 or 20 μM) HCT116 cells. β-Actin was used as internal control during western blot and qRT-PCR experiments. **(D)** Immunofluorescence was performed using FITC-labeled phalloidin, SNAI-1, SLUG, E-Cadherin, and CALD1 were stained with DAPI (Scale bar: 20 μm). Data are expressed as Mean ± SEM (*n* = 3). ^∗^*p* < 0.05, ^∗∗^*p* < 0.01, and ^∗∗∗^*p* < 0.001 versus untreated (0).

Epithelial mesenchymal transition and structural conformation in the ECM are usually associated with uncontrollable cell migration and invasion which drive metastatic aggressiveness of malignant tumor forming cells in many human cancers. We therefore examined the effect of cisatracurium on SLUG, SNAI-1, E-Cadherin (EMT marker) and CALD1 (a protein implicated in structural conformation of ECM) mRNA and protein expression levels in CRC *ex vivo* and *in vivo* by employing qRT-PCR and western blot. Analysis of the data indicates that cisatracurium exposure downregulates SNAI-1 and SLUG but upregulated E-Cadherin and CALD1 in HCT116 cells in a concentration dependent fashion (**Figures 5A–C**). Mean fold change in SNAI-1, SLUG, E-Cadherin, and CALD1 mRNA transcription level of the treatment groups (10 and 20 μM) were 0.6 and 0.17 for SNAI-1, 0.58 and 0.16 for SLUG, 3.37 and 4.17 for E-Cadherin and 2.12 and 3.66 for CALD1 of control respectively. The difference in the levels of mRNA transcription and protein expression were significant (**Figures 5B,C**).

Furthermore, immunofluorescent experiment data also indicates cisatracurium increases E-cadherin and CALD1 expression but decreases SLUG and SNAI-1 in HCT116 cells compared with control (**Figure 5D**). These observations suggest that cisatracurium inhibits EMT as well as metastasis in CRC.

### Cisatracurium Inhibits CRC Tumorigenesis and Metastatic Ability *in Vivo*

**FIGURE 6:**
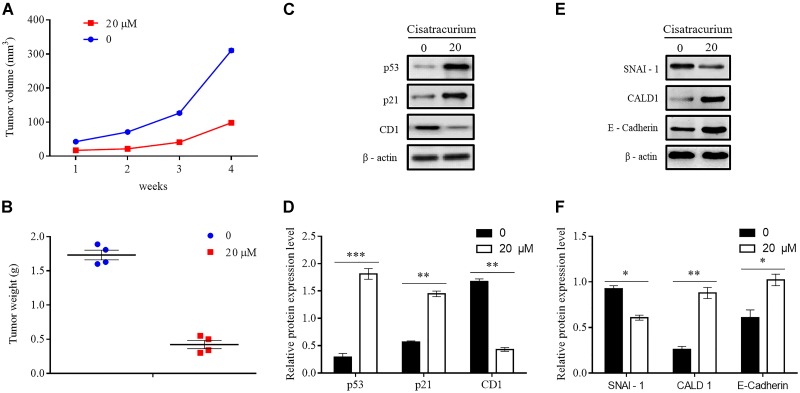
**(A–F)** Cisatracurium inhibits metastatic ability of CRC *in vivo*. **(A)** Line graph of subcutaneous tumor volume. **(B)** Weight of subcutaneous tumors in grams. Data are expressed as mean tumor volume or weight ± SE. ^∗^*p* < 0.05. **(C–F)** Representative densities of tumor viability and migration regulatory proteins (p53, p21 and CD1, SNAI-1, CALD1, E-Cadherin) in tumor tissue samples. β-Actin was used as internal control. The cluster bar chats in **(D**,**F)** indicates the levels of viability and migration regulatory proteins in the treatment group. Data are expressed as Mean ± SEM (*n* = 3). ^∗^*p* < 0.05, ^∗∗^*p* < 0.01, ^∗∗∗^*p* < 0.001 versus control.

To determine whether cisatracurium could promote inhibition of cancer development *in vivo*, a NOD/SCID mouse model was established. HCT116 cells treated with 20 μM cisatracurium for 72 h were subcutaneously inoculated into mice, and the mice monitored for 4 weeks. All the mice in both control and treatment groups developed tumors (**Figure 6A**). However, compared with the control group, treatment group had smaller tumor volume at the end of weeks 1, 2, 3, and 4. The fold increase in tumor volume in treatment group relative to control were 2 for week 2, 2.4 for week 3, and 3.4 for week 4 (**Figure 6A**). Similarly, whiles the average tumor weight in the control was 1.75 g, that of the treatment group was 0.48 g. The fold increase in tumor weight relative to that of the control was 3.65 (**Figure 6B**). These results demonstrate that cisatracurium inhibits tumorigenesis of CRC in NOD/SCID mouse xenograft model.

Apart from the overwhelming suppressive effect of the cisatracurium on colorectal malignant tumor development and growth, the cisatracurium therapy also showed significant alteration in the expression of proteins known to play key role in viability, proliferation, and metastasis in human cancers. Compared to control, cisatracurium repressed the expression of CD1 protein but elevated the expression of p53 and p21 proteins (^∗∗^*p* < 0.01 and ^∗∗∗^*p* < 0.001 versus control) (**Figures 6C,D**). Consistent with the *ex vivo* results, cisatracurium treatment decreased the expression of SNAI-1 protein but increased the expression of CALD1 and E-Cadherin compared to control (^∗^*p* < 0.05 and ^∗∗^*p* < 0.01) (**Figures 6E,F**).

## Discussion

The tumor suppressor p21 maintains genomic stability by strictly regulating the cell cycle check points (Roninson, 2002). p21 is usually deactivated in a variety of human malignancies as a result of diminished or loss of p53 activity, or loss-of-function induced by cytoplasmic translocation of p53 (el-Deiry et al., 1993; Dulic et al., 1994; Abukhdeir and Park, 2008). Bunz and Dubic respectively reported that human CRC cells lacking p21 have been demonstrated to be harboring ineffective G1 and G2 checkpoints (Bunz et al., 1998; Dulic et al., 1998). A study of p21 knockout mice exposed to gamma rays reveals a stricken accelerated tumorigenesis and wide spread metastasis (Jackson et al., 2003; Garcia-Fernandez et al., 2011). Contrarily, elevated translation of p21 protein was found to be associated with repression of melanoma development and metastasis (Genov et al., 2016).

Cyclin D1 is a multipurpose protein that controls mitochondrial function, angiogenesis and cell motility (Sakamaki et al., 2006; Wang et al., 2006; Zhong et al., 2010). Classified as an oncogene, cyclin D1 is often over-expressed in a number of human malignances including colon, breast, lung and prostate carcinomas (Arnold and Papanikolaou, 2005; Santarius et al., 2010). This is corroborated by the findings of a transgenic mice model that demonstrated that uncontrollable elevated expression of cyclin D1 is tumorigenic, and that its tumorigenicity could be mediated by mouse mammary tumor virus (MMTV), particularly in mammary hyperplasia and carcinoma development in the transgenic mice (Wang et al., 1994). It is reported that concomitant over-expression of D1 type cyclin and under-expression of tumor suppressor p21 are pre-requisite for tumor initiation, as it is evidenced that down-regulation of cyclin D1 and over-expression of p21 in xenograft model abrogate the formation of primary tumor (Dai et al., 2013). Consistent with these finding, our data showed that cisatracurium treatment up-regulates p53 and p21 expression, and decreased cyclin D1 expression. This could be attributed to the inhibitory effects of p53 and p21 that prevent the downstream intracellular signaling of cyclin D1 pursuant the cisatracurium induced genotoxic stress. We also observed that there was a significant reduction in cell growth in the cisatracurium treatment groups compared to control. The *in vivo* investigation also revealed decreased tumor growth rate in mice that were inoculated with cisatracurium treated HCT116 cells.

Tumors of epithelial origin begins as a lesion confined in an organ and eventually advance from the primary locations to invade distant secondary locations. These physiological alterations otherwise described as metastasis are reasons for the fatality of tumors. Molecular processes including EMT and debasement of the basement sheath and ECM by proteases activation have been incriminated in the process of cancer spread known as metastasis (De Craene and Berx, 2013). p53 is reported to be among the key regulators of cancer metastasis. It directly regulates the transcription of genes that mediates canonical metastasis pathways, such as anoikis, cell motility, adhesion, migration, invasion, ECM interactions, stemness, and EMT (Emily et al., 2014). Cheng et al. (2011) demonstrated that elevation of p53 in SKOV3 and PC3, known hyperinvasive human cancer cell lines, inhibited cell movement. Contrarily, depletion of p53 is implicated in uncontrollable cell migration and has been captured in a number of previous studies, where cell motility through 2D and 3D ECM-like gel were observed (Dhar et al., 2008; Junk et al., 2008; Muller et al., 2009). In addition, Dhar et al. (2008) reported wide spread metastasis of inactivated p53 cells inoculated as xenografts. Several other reports have also demonstrated that loss of p53 function alters the architectural integrity of fibroblasts, which afford them the ability to move swiftly in scratch-wound experiments and across ECM-like gel (Alexandrova et al., 2000; Guo and Zheng, 2004; Gadea et al., 2007). Consistently, the present study shows that cisatracurium induced over-expression of p53 in CRC cells and significantly attenuated cell movement through uncoated and coated ECM-like gel in concentration and time dependent fashion. In addition, the findings of this work revealed that cisatracurium treatment did not only cause over-expression of p53 but also impaired rapid cell migration in scratch-wound experiments in CRC cells.

Cells of epithelial kind are organized in an apical–basal form and are roped to adjacent cells via intercellular junctions which allow movement of only epithelial cells that are connected to one another. The motility of these cells is also incapacitated by a sheath of thin membrane, which permits only lateral movement in the epithelial stratum (Scheel and Weinberg, 2012). The processes that regulate EMT are typified by the slackening of cell–cell binding, forfeiture of epithelial morphological integrity, deficient cell polarity, and attainment of many migratory and mesenchymal phenotypes. In a well-organized scheme of events where cell to cell and cell to ECM communications get disrupted, epithelial cells are shed off from the tissues they are adhered to, permitting their cytoskeletons to be adjusted and thereby enabling them to move through the tissue (Scheel and Weinberg, 2012). EMT is activated in order to maintained this acquired mesenchymal phenotype. EMT transcription factors such as SLUG and SNAIL have been reported to be transcriptional suppressors. They repress genes unique to the epithelium, especially molecules involved in maintaining cell to cell adherent junctions, such as E-cadherin, and upregulate sections of the mesenchymal migratory system (De Craene and Berx, 2013). p53 partly impedes metastasis by negatively controlling transcription factors known to be important in initiating and maintaining EMT process (Wei et al., 2006; Chang et al., 2011). p53 signaling represses SNAIL and SLUG, which in turn increases E-Cadherin expression level to negatively control EMT (Siemens et al., 2011). Consistent with findings in this work, treatment of CRC cells with cisatracurium upregulated p53 and E-Cadherin expression but significantly inhibited the levels of SNAIL and SLUG expression compared to untreated cells in a concentration dependent fashion.

EMT is known to confer migratory potential on tumor cells, however, epithelial cells are also capable of infiltrating adjacent tissues without completely triggering EMT (Nieto and Cano, 2012). Aside its regulatory role of proteins that mediate EMT, p53 also influence the signaling axes that control ECM, cell migration, and chemotaxis which contributes to invasion and uncontrollable spread of tumor cells. Cells communicate with and migrate across the ECM via invadopodia, a cell appendage capable of triggering breakdown ECM and the thin basement connective membrane. Described as a calmodulin coupling protein, caldesmon is a protein that is code named CALD1 gene in humans (Huber, 1998). Under normal circumstances, caldesmon suppresses involuntary muscle tonicity by repressing the ATPase required to derive myosin in involuntary muscles (Wang, 2002). p53 has been reported to elevate mRNA of CALD1 and result in increased suppression of podosome synthesis which in tend lead to suppression of oncogenesis and metastasis (Mukhopadhyay et al., 2009; Wang et al., 2009). Furthermore, a recent study has shown that upregulation of CALD1 expression suppresses invadopodia/podosome formation, ECM degradation and invasion in breast and CRC cells (Yoshio et al., 2007). It has been demonstrated in this study that treatment of CRC cells with cisatracurium upregulates CALD1 expression level and inhibits cell motility and invasion. Putting together, these findings suggest that cisatracurium related up-regulation of p53 in CRC cells is associated with inhibition of transwell cell migration and invasion; repression of SNAIL and SLUG expression level, and over-expression of E-Cadherin and CALD1.

Consistent with the paradigm that p53 signaling induces cell-cycle arrest, senescence, or apoptosis when cells are exposed to various forms of stress, including DNA damage, we hypothesized that the increase in the level of p53 expression in cisatrcurium treated CRC cells could be due to DNA damage. To investigate this hypothesis, comet assay was employed to detect the presence of DNA damage in untreated and treated CRC cells. Our results revealed that cisatracurium causes DNA damage, and the degree of DNA damage is proportional to the concentration of cisatracurium. This finding seems to suggest that cisatracurium-induced p53 up-regulation, confers a corresponding downstream effect on both proliferation and metastasis related genes and proteins, and could be a response to the observed DNA damage.

## Conclusion

The findings of this work suggest that elevation of p53, p21, E-Cadherin, CALD1 with downregulation of CD1, SNAI-1 and SLUG upon DNA damage, following cisatracurium exposure represent the potential mechanism by which cisatracurium causes the inhibition of CRC cells/tumor growth, and regression of migration and invasion. Therefore, in the light of intense search for anesthesia technique devoid of cancer metastasis and recurrence risk for tumor resections requiring adequate muscle relaxation or continuous controlled ventilation, these findings hold great prospects.

## Author Contributions

QW and Z-LL designed the study. MI assisted in the study design. IY, JS, and JH performed the experiments. IY and JS analyzed the data. IY and QW prepared the article. MI and WW revised the manuscript. All the authors read and approved the final article for submission.

## Conflict of Interest Statement

The authors declare that the research was conducted in the absence of any commercial or financial relationships that could be construed as a potential conflict of interest.

## Abbreviations

CCK-8: Cell Counting Kit 8
CRC: colorectal cancer
FBS: fetal bovine serum
HCT116: human colorectal carcinoma cell line
PBS: phosphate buffer solution
qRT-PCR: quantitative real time -polymerase chain reaction
SDS-PAGE: sodium dodecyl sulfate-poly acrylamide gel electrophoresis
